# California State Dental Association

**Published:** 1872-09

**Authors:** 


### CALIFORNIA STATE DENTAL ASSOCIATION.

### Proceedings of Third Annual Meeting.

### FIRST DAY—MORNING SESSION.

The third annual session of the California State Dental Association convened in Mozart Hall, San Francisco, at 10 o'clock A. M., June 11th, 1872, the President, Dr. S. W. Dennis, in the chair.

All the officers were present at roll-call except First Vice President Pierson, who was absent from the State.

All the Standing Committees and six of the seven essayists announced themselves "ready to report when called upon."

Dr. S. M. Harris, of Grass Valley, and George McCowen, D. D. S., of Ukiah City, were admitted to membership.

After the usual preliminary resolutions, regulating hours of meeting, adjourning, etc., adjourned to 2 o'clock P. M.

### AFTERNOON SESSION.

S. E. Knowles, M. D., and D. M. Hughes, of San Francisco, were admitted to membership. The President then delivered the "Annual Address."

He spoke in eloquent terms of the worthy efforts being made by the members of the association, to place our profession side by side, if not in advance, of the other learned professions of the age; and how the profession on the Pacific coast had for twenty years floated about like a deserted ship, that it had been watched by some members of the profession, and was at last boarded and manned by a crew that would sail her triumphant under easy but taut canvas—and, that if all who shipped with us do their duty, we will soon so extend our sphere of usefulness, as we are borne along on the journey of professional life, as to command the respect of those rich with intellectual culture, social and moral worth, and, in fact, the whole civilized world. He spoke at length of the necessity of a thorough dental education, regretting that the several dental journals, which "have done more for the advancement of the profession than other medium," were so little read and supported by dental practitioners. If all understood their duty, every important truth would find its way into said journals, and the journals would find their way into every dentist's office.

Of the "Rubber Dam" he said: "The vehicle of thought is made futile to the magnitude of this question. Language is as helpless as the fœtus in embyro, for too much can not be said in praise of, or done for the inventor, Dr. S. C. Barnum, for his generous gift of it to the profession." Believed there was no dentist worthy the name who did not owe much for his professional status to associated effort. Urged the necessity of harmonious intercouse, interchange of thought and discussion—using all our talents to make our association acceptible to God, commendable to man, and subservient to the wants and necessities of the human race.

The address was received with applause. Referred to Publication Committe.

Wm. Dutch, D. D. S., Chairman of Committee on Operative Dentistry, made an able written report, referring to latest improved materials and instruments in use in this department. Said that properly prepared cavities should be as free from undercuts as possible, all fissures thoroughly excavated, and orifice thoroughly smoothed to make perfect joints. Soft foils were not indispensable, yet most difficult operations can be safely undertaken with cohesive foils by the faithful operator. All would fail when used by the *unfaithful*. Of heavy foils, prefers the *rolled* to the *beaten*. Urged perseverance in the application of the "Rubber Dam," as it well repays for all time and trouble, as success is attained with, where failure is certain without it. Considered the burring engines indispensable to the operating room.

Discussion—In answer to a question, Dr. Dutch said he never sacrificed any portion of the teeth that could be saved. Protects weak walls by lapping the gold over them to prevent their being forced away by antagonizing teeth or other causes.

Dr. Knowles used soft light foils—No. 4 principally—against frail walls to avoid the difficulties of imperfect adaptation, incurred by the heavy; the heavy leaving minute angles, creases and crevices; generally finished up with the heavy foil. Influenced by circumstances. Very seldom uses retaining pits. In contour filling uses thread-cut wires to build around—anchoring them in the dentine, sometimes as many as three in a tooth, thus retaining fillings securely that could not be by the ordinary *pit*. Always finds space to insert them in "*living teeth*" between pulp and enamel, without injury to pulp. Advocates smooth folding of foil.

Dr. Roberts "*Crimps*" his foil—is not afraid of injuring it by contact with *clean* figures.

All agreed that fine points frosted or slightly serrated were best for adhesive gold.

Drs. Prather and Plomteaux used the *broken points* spoken of in March No. of *Missouri Dental Journal* almost universally; believed that none who tried them would use any others.

J. J. Menefee, D. D. S., read an essay on "The Pathology and Treatment of Alveolar Abscess." Discussed by Dennis, McCowen, Dutch and Knowles.

The President announced that Dr. Dutch would give a clinic at 1 P. M. to-morrow, in Operative Dentistry

Adjourned to 8 o'clock P. M.

EVENING SESSION.

Dr. Knowles made verbal report on the "Causes of Irregularities of the Teeth and Best Methods of Correcting the Same;" exhibiting models of a large number of difficult cases that had been under his treatment, also the appliances used in their correction, conclusively showing that the most difficult cases can be improved, if not entirely overcome, by skillful treatment and perseverence. Believed one of the principal causes of irregularity is that the child inherits the teeth of one parent, and jaws of the other. Much injury is likely to result by too rapidly moving the teeth when correcting irregularities. The mouth should be kept in as healthy condition as possible during the operation, by aid of such remedies as each case may demand.

Dr. Sichel read report on Dental Chemistry. Used as a substitute for red vermillion, in vulcanite, the following preparation: Dissolved oxide of iron in nitric acid: put in crucible and anneal to smelting heat; cool, pulverize, and mix with the rubber.

Dr. Austin made verbal report on Dental Pathology and Surgery.

Adjourned to 10 o'clock A. M., to-morrow.

### SECOND DAY—MORNING SESSION.

Officers present same as yesterday.

Drs. Woodward, Bush and Myers, of Committee on Mechanical Dentistry, each made written report. Reported no important improvements. Believed gold in skillful hands a *good and proper* base at all times, especially in partial sets. Allen's continuous gum work secures the most correct expression and articulation, and is very desirable for entire sets.

Said reports were discussed at length.

Dr. Cool generally used vulcanite, metal lined, especially all partial inferior sets with Nos. 18 or 19 alluminum. Plates thus lined never gave way.

Dr. Dennis believed any material improperly manipulated would produce irritation by mechanical action; believed rubber had been injurious to the profession on account of its wholesale use by incompetent and dishonest operators.

Dr. Plomteaux did not believe in condemning any material simply because quacks used it; would advocate the use of any material that had merit in it, when properly manipulated.

Dr. C. C. Knowles had not the slightest doubt but that gold work when properly adjusted would answer every purpose of mechanical dentistry. Believed vulcanite far superior to any other cheap base extant, and that it had been a great blessing to the world. "That it had been a curse to the profession, we may well entertain different opinions." Does not believe there is a well authenticated case on record of its noxious character, or injury to the mouth, when properly cured and manipulated.

Adjourned to 3 o'clock P. M.

During the recess Dr. Dutch gave a very interesting clinic before the society. Operation, excavating, and filling complicated cavity in superior molar.

### AFTERNOON SESSION.

Drs. Plomteaux, Knowles, Knox, Menefee and Younger were appointed a committee to report at next annual session what they deem expedient regarding State legislation for the regulation and practice of dentistry. On motion Dr. Dennis was added to the Committee.

Dr. Knox, Chairman of Committee on Dental Literature and Education, read report; and Dr. Birge read an essay on "The Causes of Degenerancy of the Teeth." Said papers contained much of merit, but owing to lateness of the hour and press of business were not discussed.

A vote of thanks was tendered Dr. Dutch for his interesting and efficient clinic, being the first on Operative Dentistry given before the society.

Dr. Knox nominated Profs. J. Foster Flagg, of Philadelphia, and Wm H. Atkinson, M. D., of New York; also Dr. Geo. A. Mills, of Brooklyn, N. Y., as honorary members; unanimously elected.

Dr. Crosset exhibited a pathological specimen, consisting of a fibrous tumor that had been recently removed from the mouth of an inhabitant of San Francisco.

Adjourned.

EVENING SESSION.

Dr. Crossett, of Committee on Dental Pathology and Surgery, read an interesting paper which was received with applause.

Drs. Henry C. Davis and Wm. S. Davis, of San Francisco, were admitted to membership.

Dr. Plomteaux read an essay on "Dentistry," its history, present status, claims and relations.

(The Secretary has ommitted to say what should be said of his own paper, that it was a very ably written essay. He handled the subject assigned to him in a masterly manner, which elicited much favorable comment—*Publishing Committee.*)

Adjourned to 10 o'clock A. M. to-morrow.

THIRD DAY—MORNING SESSION.

President Dennis in the chair.

Committee on Publication moved indefinite postponement of the subject referred to them at our last annual session regarding publication of dental periodical under auspices of this society; carried.

Dr. Knox read essay on Dental Therapeutics.

The President gave notice that Dr. Knox would give a clinic at 1 P. M., to-day, on Filling; and clinics by Dr. Dennis, on Administration of Nitrous Oxide Gas and Extracting, at 9 A. M., to-morrow; and by Dr. Sichel, at 10 A. M., on Preparation of Oxide of Zinc.

Drs. Plomteaux, Knowles, and Knox, were appointed to revise the Constitution, By-laws, and Order of Business, and to prepare a code of ethics and rules of order; to be considered at next annual session.

Vote of thanks tendered to the several railroad companies, for half-fare rates to members.

Dr. Dutch, delegate to the American Dental Association, spoke at length of the recommendation of said body, relative to formation of local societies, and necessity of appropriate instruction to the profession generally. A long and interesting discussion followed, on the elevation and education of the profession, and the necessity of educating the public up to the necessity of paying more attention to the subject of dentistry.

Adjourned to 2 o'clock P. M.

AFTERNOON SESSION.

Dr. N. Shrewsberry, of Santa Barbara, was admitted to membership.

Dr. Younger read essay on "The Causes and Treatment of Loose Teeth." He generally removed the *cause* of loose teeth, and treated surrounding part with tincture of iodine. Did not believe in the use of acids for removing tartar. Preferred fine instruments; if skillfully used will not injure the periostium.

Dr. Knowles prefers chloride of zinc to tincture of iodine; believes it more of a stimulant to the soft parts.

Dr. Dennis treats similar to Dr. Younger; has frequently reproduced the gums by said treatment.

Dr Dutch, on behalf of the San Francisco Dental Association, invited the State Association to dine with them at 6 o'clock P. M., to-morrow. The invitation was accepted.

Corresponding Secretary Knox read letters from Profs. Judd, Taft, and McQuillen.

Dr. Dennis, on behalf of the author, presented the Society with a copy of "The Claims of Dentistry," by Oliver Wendell Holmes, M. D.

Dr. Dennis read a highly interesting paper, entitled: "What Makes Teeth Decay.

Dr. Knox was tendered a vote of thanks for his efficient clinic, as per appointment to-day.

EVENING SESSION.

The following officers were elected for the ensuing year.

President—Wm. Dutch, D. D. S., of San Francisco; First Vice President—J. J. Meenfee, D. D. S., of San Jose; Second Vice President—Dr J. N. Meyers, of Stockton; Third Vice President—Dr. S. M. Harris, of Grass Valley; Corresponding Secretary—Henry E. Knox, D. D. S., of San Francisco; Recording Secretary—Dr. H. J. Plomteaux, of Woodland; Assistant Recording Secretary—Dr. L. G. Yates, of Centerville; Treasurer—Dr. C. C. Knowles, of San Francisco; Libarian—Dr. J. J. Birge, of San Francisco.

The newly elected officers took their stations, and entered at once upon the discharge of their duties.

The *usual* vote of thanks was extended the retiring officers for efficient services during their term, especially to Ex-President Dennis, for his able and highly satisfactory administration.

Adjourned to 10 o'clock A. M., to-morrow.

FOURTH DAY—MORNING SESSION.

President Dutch in the chair. Officers all present except First and Second Vice Presidents.

Dr. H. W. Calvert, of San Francisco, was admitted to membership.

By request of the association, Dr. Roberts read correspondence in his possession relative to the death of Mrs. O'Shaughnessy, under the administration of nitrous oxide gas, in New York City, March 20th, 1872.

The subject of anæsthetics and their use was thoroughly discussed, and the following resolution adopted as the sense of the association:

*Resolved*, That we, as a body, discountenance the indiscriminate use of anæsthetics for the extraction of teeth; and furthermore, that when such agents are necessary, their administration should not be undertaken by any one not thoroughly acquainted with their influence upon the patient, and having always at hand all the best-known restorative agents.

By invitation of the association, Dr. Crossett read a paper on Dental Literature and Education. The following resolution *apropos* of the subject was referred to the Committee on Dental Literature and Education, to be reported on at next annual session:

*Resolved*, That, as a dental association, we will secure the columns of some public print (if it can be done at a reasonable expense), by which all members of the association may have a medium through which information may be diffused amongst the mass of our population within this State; all articles for publication to be supervised by the Publication Committee.

The President announced that Dr. C. C. Knowles would give clinics at his office at 1 o'clock P. M.—Operations Filling and Extracting. Adjourned.

AFTERNOON SESSION.

Drs. S. E. Knowles, H. H. Pierson, S. H. Roberts and S. W. Dennis, were elected delegates to the American Dental Association; and the President and Secretary were authorized to furnish certificates of election to such members as would attend, until the full number to which we are entitled shall be appointed.

On motion of Dr. Dennis, a committee of five was appointed to devise ways and means of securing a suitable testimonial to Dr. Barnum for the free gift of his valuable invention—*the rubber dam*—to the profession. Drs. Dennis, Austin, Knowles, Meyers and Menefee were appointed, and by vote Dr. Dutch was added.

Resolution of thanks to Drs. Dennis, Knowles and Sichel for their instructive clinics before the society to-day, as per appointments, unanimously adopted.

The President appointed the following Standing Committees for the ensuing year:

On Arrangements—Drs. Austin, Sichel, Klein, Myers and Cogswell.

On Publication—Drs. Dutch and Plomteaux (ex-officio), Knox, Dennis and Bunnell.

On Operative Dentistry--Drs. Dennis, Pierson and Woodward.

On Dental Chemistry—Drs. C. C. Knowles, Prather and Belle.

On Dental Pathology and Surgery—Drs. Younger, S. E. Knowles, Harris, Kingsbury and Wilbirt.

On Mechanical Dentistry—Drs. Menefee, Spencer, McCowen, Bush and Calvert.

On Dental Literature and Education—Drs. Crossett. Cutlar, Yates, Eaton and Lundborg.

On Auditing—Drs. Birge, Kingsbury and Cool.

It was resolved to hold the next annual session at San Francisco, commencing on the third Tuesday in May, 1873, at 10 o'clock, A. M.

Dr. Shrewsbury recommended the following formula, which he claimed would entirely "paralize the nerve in a very short time," the only originality claimed being the *proportions* of its ingredients. In many cases when using the several agents for destroying nerves, the cauterizing gets the start of the paralyzing element, which never occurs with this preparation:

R. Arsenicum gr. x.
Tannin gr. xv.
Sulph. Morphia gr. xx.
Creosote gtt. x.

The President was given one month's time to select subject for theses, and make assignments for next annual session.

An invitation was given to the reporters of the press to be present at the dinner, at 6 o'clock P. M.

The association then adjourned its third annual session.

H. S. Plomteaux, Recording Secretary.

From the San Francisco *Daily Morning Call*, of June 15th, 1872: "The association now consists of fifty-two members, and will compare favorably with any similar body in the United States. * * * * At Martin's restaurant, in the evening, the President of the San Francisco Dental Association, Dr. Knowles, took the chair, and made a few appropriate remarks as to the welcome of the State association to San Francisco by their brother dentists of the San Francisco Association. * * * After the dinner had been disposed of, many toasts were given and responded to; everything tending to promote good-will and unity among those gentlemen who make dentistry their study. The party separated about 11 o'clock.

